# Recent Trends and Applications of Molecular Modeling in GPCR–Ligand Recognition and Structure-Based Drug Design

**DOI:** 10.3390/ijms19072105

**Published:** 2018-07-20

**Authors:** Xiaojing Yuan, Yechun Xu

**Affiliations:** 1 CAS Key Laboratory of Receptor Research, Drug Discovery and Design Center, Shanghai Institute of Materia Medica, Chinese Academy of Sciences (CAS), Shanghai 201203, China; xjingyuan@simm.ac.cn; 2 School of Pharmacy, University of Chinese Academy of Sciences, Beijing 100049, China

**Keywords:** GPCR, receptor–ligand recognition, drug design, molecular modeling, molecular dynamics, docking, binding affinity, binding pathway

## Abstract

G protein-coupled receptors represent the largest family of human membrane proteins and are modulated by a variety of drugs and endogenous ligands. Molecular modeling techniques, especially enhanced sampling methods, have provided significant insight into the mechanism of GPCR–ligand recognition. Notably, the crucial role of the membrane in the ligand-receptor association process has earned much attention. Additionally, docking, together with more accurate free energy calculation methods, is playing an important role in the design of novel compounds targeting GPCRs. Here, we summarize the recent progress in the computational studies focusing on the above issues. In the future, with continuous improvement in both computational hardware and algorithms, molecular modeling would serve as an indispensable tool in a wider scope of the research concerning GPCR–ligand recognition as well as drug design targeting GPCRs.

## 1. Introduction

G protein-coupled receptors (GPCRs) constitute one of the most important membrane families and are the target of more than 30% US Food and Drug Administration (FDA)approved drugs [1]. They are thus hot spots for both academic research and industrial innovation. Characterized by a common seven-transmembrane domain, GPCRs are capable of translating extracellular signals into the intracellular side. The signals are usually initiated by binding of extracellular ligands, including hormones, neurotransmitters, chemokines, drugs, and so on, to the receptor. Upon ligand binding, GPCRs change their conformations to stimulate different downstream signaling pathways [2]. The underlying mechanisms are highly complicated, and the progress of related studies has already been summarized in other review papers [2,3,4,5]. As the basis of the signal transduction, the understanding of receptor-ligand recognition is of paramount importance.

Molecular modeling serves as a computational microscope to systematically explore molecular structure and dynamic behavior, revealing the underlying mechanisms at experimentally unreachable spatial and temporal scales [6]. Modeling methods are now widely used to investigate the structure, dynamics, and thermodynamics of biological systems. The types of biological activity that are usually inspected using molecular modeling include enzyme catalysis, function-related conformational changes of macromolecules, and molecular recognition between small molecules and proteins or DNA [6,7,8]. Here, we will concentrate on recent advances in the use of computational modeling methods in the study of GPCR–ligand binding mechanisms and their applications in the design of drugs targeting GPCRs.

## 2. Using Molecular Modeling in the Investigation of Mechanisms Underlying GPCR-Ligand Recognition 

### 2.1. Decoding the Mechanism of Ligand-GPCR Binding Using MD Simulations

Molecular recognition is one of the most important events in molecular biology, forming the basis for the folding of proteins, the specificity of enzyme catalysis, the pathways of signal transduction, etc. [9]. It thus offers great opportunities for using exogenous ligands that could bind to a specific macromolecular target to modulate biological systems. Accordingly, understanding the mechanism underlying ligand-GPCR recognition is fundamental to discovering medications for related diseases or finding probes to elucidate unknown biological functions of GPCRs.

With the development of structural biology techniques, including X-ray crystallography, nuclear magnetic resonance (NMR) and cryo-electron microscopy (cryo-EM), it becomes possible to determine the bound pose of a ligand interacting with GPCR. However, the thermodynamic and kinetic mechanisms underlying the ligand-receptor recognition process are important but missing in the determined complex structures. Molecular dynamics (MD) simulation is a computational technique that can simulate the structural dynamics of macromolecules over time [10]. In the past decades, MD simulation has shown immense potential in providing insights into both thermodynamic and kinetic mechanisms of ligand-GPCR binding. 

Ligand binding events occur on a timescale ranging mostly from nanoseconds to microseconds, which in the past were too slow to be captured by unbiased MD simulations [11]. Owing to the advances in both computational hardware and algorithms, such as the recent development of supercomputers [12] and the implementation of MD codes for GPUs [13], it is now possible to simulate the spontaneous association of ligands with GPCRs by use of unbiased MD. In the work by Dror et al., entire processes of several drugs binding to two archetypal GPCRs, β_1_- and β_2_-adrenergic receptors (β-ARs), were captured by multiple-microsecond timescale MD simulations [14]. The dominant binding pathways are similar and start from the drugs contacting a vestibule on the extracellular surface of the receptors. Then drugs squeeze from a narrow tunnel, formed by the extracellular loops (ECLs) and helices of GPCRs, to move into the final binding sites. The on-rates of the drugs were also estimated and were found to be related to the dehydration of ligands and the binding pockets. In a following study from the same group, the binding sites and specific drug–receptor interactions of several allosteric modulators of M2 muscarinic acetylcholine receptor (M2 mAChR) were also investigated using unbiased MD [15]. With the help of these findings, rational structural modifications were successfully achieved to enhance a modulator’s allosteric effects. 

The unbiased MD simulations presented above are resource-consuming, and more advanced information regarding the free energy landscape underlying the ligand binding could not be obtained. Enhanced sampling methods, such as steered molecular dynamics, metadynamics (MetaD), and accelerated molecular dynamics (aMD) can acquire the desired information at a moderate computational cost. MetaD is capable of enhancing conformational sampling and subsequently reconstructing the free energy surface (FES) as a function of a few selected degrees of freedom, called collective variables (CVs) [16]. Recently, this method and its variants have proven to be effective in studying ligand-GPCR recognition [17,18,19,20,21]. Using MetaD simulations, Provasi et al. investigated the mechanism of an antagonist binding to the delta opioid receptor [19]. On the basis of the MetaD trajectories, an energetically favorable binding pathway was proposed in which the ligand firstly moves into a cleft formed by the ECLs of the delta receptor before entering its final binding site, which is reminiscent of the dominant pathway of β-ARs-ligand association revealed by unbiased MD simulations. AMD adds a non-negative boost potential to the system’s potential energy so as to accelerate transitions across the low-energy states [22]. A similar recognition mechanism has also been depicted in an aMD study of ligands binding to a mAChR in which all the tested ligands were observed to associate with the extracellular vestibule before entering the final pocket [23]. In summary, the association of ligands with the extracellular vestibule is a common metastable state along the pathways of ligand binding to GPCRs from the solvents. 

Other than binding directly from the water phase into sites inside the extracellular crevice of GPCRs, some ligands reach their binding pockets via the membrane environment. Hurst et al. performed unbiased MD simulations to capture the access of sn-2-arachidonoylglycerol (2-AG) to the CB2 cannabinoid receptor via the lipid bilayer and provided a qualitative description of the key steps along the binding pathway [24]. This result suggests that 2-AG first partitions out of the bulk lipid and then enters the binding pocket through the interface between TM6 and TM7. The Markov state model (MSM), which is capable of extracting system dynamics from aggregates of short MD trajectories, has lately been utilized in a research of the lipid-involved ligand binding pathway [25]. Using distributed computing on the GPUGRID combined with MSMs [26], Stanley et al. performed over 800 µs MD simulations to quantitatively interpret the binding of a lipid-like inhibitor, ML056, to the sphingosine-1-phosphate receptor 1 (S1P1R) [27]. The resulting pathway resembles Saleh’s finding in which lipid-like ligands bind to the intra-helical site of GPCRs through two steps, partitioning out of bulk lipids to reach a “membrane vestibule” and then accessing the final binding site by passing through the channel formed by transmembrane helices. Moreover, they found that the entry of ML056 into the “membrane vestibule” is the rate-limiting step of the whole binding process.

In addition to these lipid-involved pathways for ligand binding to the intra-helical site of GPCRs, we recently described the association of an antagonist with an extra-helical GPCR binding site located at the interface of the membrane and the protein. We will reflect on this point in the following case study.

### 2.2. The Role of the Membrane in Ligand-GPCR Recognition

As a representative family of membrane proteins, the role of the membrane in the recognition of GPCRs with their ligands has gained increasing attention. Several models have been proposed to address this issue, including the microkinetic model, the reduction in dimensionality model, the rebinding model, etc. [28,29].

According to the microkinetic model, the concentration of hydrophobic ligands in the membrane solvated layer may be boosted due to their intrinsic affinity to the membrane, and the ligand occupancy around the receptor is thus increased. This is in line with observations from several MD simulations investigated on small hydrophobic molecules trafficking inside the lipid bilayer [21,30]. Consequently, the experimentally measured ligand binding affinity may be overestimated compared to its “actual” affinity to the receptor [28,29]. This assumption has been verified by investigating the interactions of several ligands with β_2_-AR using both molecular docking and computational solvation analysis [31]. It is thus important to deconvolute membrane–ligand and GPCR–ligand binding contributions to gain a more reasonable interpretation of the structure–activity relationship (SAR) and efficiently guide hittolead evolution.

The “reduction in dimensionality” effect presumes that ligands reach the receptor via two-dimensional diffusion within the plane of the membrane so as to increase the ligand–receptor collision rate. The rebinding model proposes that ligands may undergo multiple “binding–unbinding” events with the receptor before drifting away from the membrane, and a long “overall” residence time of the ligands could be acquired. These kinetic effects are important for drug design but difficult to test by general experimental techniques, so this is exactly where molecular modeling methods can serve as superb alternatives. 

### 2.3. Case Study 1: The Mechanism Underlying an Antagonist Binding to the Extra-Helical Site of P2Y_1_R

In this case study, we investigated the recognition mechanism of an antagonist with the extra-helical site of a GPCR using multiple computational modeling approaches [21]. The P2Y_1_ receptor (P2Y_1_R) is a class A GPCR activated by ADP to induce platelet activation and is a promising target for the design of novel antithrombotic drugs. Recently, the crystal structure of P2Y_1_R bound with its non-nucleotide antagonist, 1-(2-(2-(tert-butyl) phenoxy)pyridin-3-yl)-3-(4-(trifluoromethoxy)phenyl)urea (BPTU), was reported. Notably, the extra-helical binding site of BPTU is located in between the receptor and the lipid bilayer [32]. It is interesting to see how BPTU finds its way to such a unique binding site.

**Figure 1:**
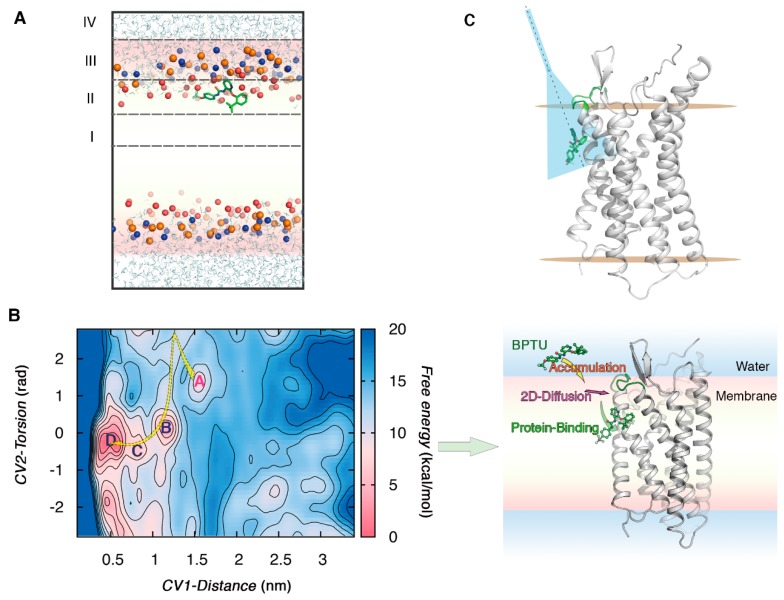
The mechanism of BPTU binding to the receptor–lipid interfacial pocket of P2Y_1_ purinergic receptor (P2Y_1_R) investigated by multiple computational simulation methods. (**A**) 1-(2-(2-(tert-butyl) phenoxy)pyridin-3-yl)-3-(4-(trifluoromethoxy)phenyl)urea (BPTU) molecules spontaneously penetrate and stay in Region II of the bilayer in unbiased molecular dynamics simulations. (**B**) The free energy surface (FES) underlying the BPTU–P2Y_1_R association process and the proposed energetically favorable binding pathway. Four major energy minima found on the FES were labeled A–D. (**C**) The diagram of the funnel metadynamics simulation.

To study the mechanism of ligand binding to a lipid-exposed site, it is crucial to understand how the ligand partitions and localizes in the lipid bilayer. We first conducted two 500-ns conventional MD (CMD) simulations, each starting with one BPTU molecule being positioned in the aqueous phase. In both simulations, BPTU quickly penetrates the bilayer within 50 ns, until it arrives at the interface of the polar and lipophilic region of the bilayer (Region II) (Figure 1A). To provide a more quantitative view, the free energy of transferring BPTU from the center of the bilayer to the water phase was calculated using the umbrella sampling method. The results show that there is no energy barrier to BPTU entering the lipid bilayer and the energy minimum corresponds to the position of BPTU near the center of Region II. Accordingly, Region II may serve as a reservoir for BPTU before it reaches the interfacial pocket on the receptor. This is in line with the microkinetic model that a favored region of the bilayer accommodates the small molecules and acts as a depot to accumulate ligands around the receptor.

Next, we investigated the mechanism of BPTU binding to this special site using metadynamics-based methods. We performed a well-tempered metadynamics (WT-MetaD) simulation to simulate the complete BPTU–P2Y_1_R association process. Three CVs that have been widely used in previous studies on ligand binding were utilized in our simulation: (1) the distance between BPTU and the binding site; (2) the torsion angle between BPTU and residues in the pocket; (3) the coordination number between BPTU and the Cα atoms of the receptor residues. Then, we constructed the free energy surface (FES) underlying the binding process, using CV_1_ and CV_2_ as reaction coordinates. It was revealed that the binding includes three key steps: BPTU first enters from aqueous solution to Region II of the bilayer, then diffuses in Region II to interact with the second extracellular loop (ECL2), and finally penetrates into the lipid–receptor interface to reach the target site (Figure 1B).

The FES depicted above gives us a general view of the ligand–receptor recognition; however, the binding affinity could not be calculated accurately due to the lack of recrossing events between the bound and unbound states. Therefore, the funnel metadynamics (FM) method [33] was used to limit the region and increase the efficiency of sampling (Figure 1C). The FM trajectory lasted 0.75 µs, in which BPTU was released from the crystal bound state, diffused out of the membrane, and finally explored the unbound states solvated in the solvent. Several recrossing events were achieved, resulting in a quantitatively well-characterized FES and an accurate estimation of BPTU–P2Y_1_R binding affinity. The calculated binding free energy is in exceptional agreement with the experimental results (∆*G*_b0_calc_ = −11.5 kcal mol^−1^, ∆*G*_b0_exp_ = −11.7 kcal mol^−1^). These findings broaden our understanding of the ligand–GPCR binding in the membrane environment and provide a vivid example of the related theoretical models. 

## 3. Applications of Molecular Modeling in Structure-Based Drug Design of GPCRs 

Computational modeling has played a crucial role in the discovery of many marketed drugs and drug candidates [34,35,36,37,38,39,40,41]. Nowadays, structure-based drug design (SBDD) and ligand-based drug design (LBDD) in computational forms have become core components of modern drug discovery [42]. They differ in terms of whether a 3D structure of the target is used in the design process. LBDD utilizes the structure and activity data of ligands determined by experimental methods. It depends on the substantial activity of ligands, and the novelty of the discovered compound structures is rather limited. In contrast, SBDD can be carried out with the available 3D structure of the target, and active chemicals with novel scaffolds have been discovered with this strategy.

Since the invention of the first docking program in the early 1980s [43], at least three classes of computational SBDD methods have been developed. The molecular docking methods including DOCK, AutoDock, GOLD, Glide, FlexX, ICN, etc., which are integrated in either an open-source or a commercial program, were widely used to predict the protein–ligand interactions. The second class mainly includes approximate free-energy calculation methods such as the linear interaction energy (LIE) [44] and the MM-PBSA/GBSA [45,46,47,48] methods, in which the motion of both the solvent and the protein are considered in the free energy calculation with some approximations. The third is free-energy calculation methods that are used to calculate the relative or absolute binding free energy. The relative binding free energy methods, also termed alchemical calculations, use MD simulations to completely sample and then compute binding free energy differences between structurally similar ligands [49]. The absolute binding free energy methods are the most computationally expensive but powerful approaches [50,51,52]. Without any prior knowledge of the binding affinity of related ligands, absolute binding free energy methods use MD to extensively sample the ligand–receptor recognition process and subsequently predict the bound pose, binding affinity, and other thermodynamic properties. 

The next subsection discusses in detail the application of two methods, molecular docking and free-energy calculation methods, which are the most frequently used and the fastest growing methods, respectively, in structure-based design of drugs/probes targeting GPCRs.

### 3.1. The Most Widely Used Method: Molecular Docking 

Docking is the fastest among all the aforementioned approaches for predicting protein–ligand interactions. It is often the choice for screening large compound libraries in a relatively short time, which is desirable in hit identification during the early stages of drug discovery. This kind of method also contributes to lead optimization and even the analysis of drug metabolism when using P450 isoforms as target structures [51]. Consequently, molecular docking is one of the most frequently used methods in GPCR SBDD programs. We summarize recent representative docking campaigns targeting GPCRs in Table 1.

In general, docking starts with compounds from databases and 3D structures of the target to generate the proper ligand–protein binding conformation and to rank the bound poses using scoring functions [68]. The workflow, scoring functions, and software of docking have been reviewed [68,69,70,71]. The 3D structure of the target is fundamental to the success of docking, so next we will specifically discuss widely used methods for acquiring GPCRs’ 3D structures. Due to the tremendous strides made in structure determination techniques, including NMR, X-ray crystallography, and Cryo-EM, the number of available 3D structures of GPCRs is increasing rapidly [72]. There have been several discovery campaigns in which large-scale compound libraries were docked to experimentally determined structures of class A GPCRs, and nanomolar-affinity ligands with distinct scaffolds have been identified [56,60,61,62,63,64,65,67].

Owing to the difficulty of structure determination, in many cases the structural information of GPCRs is still largely limited, and no 3D structure is available. The solved GPCR structures have already been systematically reviewed elsewhere [73,74,75]. Here, we will introduce several computational modeling methods frequently used in generating 3D models of GPCRs, from those that require the least computational resources to those that require the most. When the sequence similarity between the target structure and the homologous one is high enough (usually over 30% sequence identity), homology modeling is used to determine the 3D structures of GPCRs. The general idea behind the homology modeling (HM) method is that 3D structures of proteins are more conserved than their sequences [76]. Thus, proteins with homologous sequences have similar 3D structures. Since the determination of the first GPCR crystal structure in 2007, HM has proven effective in providing 3D models of GPCRs that serve as the basis for SBDD [52,53,56,57,58,59,62,66,77].

However, HM would not be a recommended method when the sequence similarity between the template and the target is poor or the number of available template structures is limited. More advanced modeling techniques such as ligand-supported HM approaches are needed. The conformation of the ligand binding pocket could be optimized by taking the information of the known compounds into account. In the work by Nowak et al., 400 3D models of 5-HT_1A_ were generated and docked against known ligands [77]. The ligand-bound conformations were used to select and improve the models of the receptor. Ultimately, the quality of the tuned model was evaluated by docking it with known 5-HT_1A_ ligands and decoys. Evers et al. developed a similar approach named MOBILE (Modeling Binding Sites Including Ligand Information Explicitly) and successively applied it to discovering antagonists of neurokinin-1 (NK-1) and alpha1A receptors [53,55,78]. Diverse compounds with sub-micromolar binding affinity were found. It is noteworthy that the success of MOBILE depends on the agreement between the model and the template structure in the ligand binding site. If the differences between them are huge, additional experimental information about the binding pocket, such as mutagenesis data, is required. The ligand-supported HM approaches have been used in several docking campaigns to identify bioactive compounds against GPCRs with limited structural information of receptors [69,79,80,81,82]. We will describe in detail the representative research in the following case study.

The PREDICT algorithm [83] is a de novo approach for modeling the 3D structures of GPCRs based on their sequences and the structural constraints imposed by the membrane environment. Unlike the aforementioned HM techniques, which rely on known homologous structures, PREDICT centers on the physicochemical properties of the receptor’s primary sequence. This algorithm optimizes thousands of alternative conformations at the same time to ensure the most stable one is found. Becker et al. applied PREDICT to generate GPCR models for the discovery of active compounds targeting six different GPCRs, which include the serotonin 5-HT_1A_, serotonin 5-HT_4_, dopamine D2, neurokinin NK1, neuropeptide Y Y1, and chemokine CCR3 receptors [54,84]. In these virtual screening studies, more than 100,000 compounds were initially docked to each target, and in vitro binding assays confirmed 12–21% hit rates. These results reveal the capability of PREDICT to model 3D structures of typical GPCRs used as a starting point of SBDD.

In some cases, the quality of docking could be further improved by taking account of the receptor’s flexibility using induced fit docking (IFD) [85,86] and ensemble docking methods [87]. IFD is often used to model the flexibility of a side-chain or even a backbone of amino acids involved in the ligand binding site [88]. The ensemble docking method utilizes more than one 3D structure of the receptor, usually generated by MD simulations. Recently, more advanced MD-based methods have been used to generate the starting point for ensemble docking. Miao et al. introduced aMD simulations into the virtual screening workflow to build the ensemble of mAChRs conformations [89]. Retrospective docking of known ligands and decoys to mAChRs by use of the workflow with or without the aMD-generated conformation ensemble of the receptor shows that the application of aMD significantly improves the enrichment factor of the overall workflow. With the help of IFD and aMD-augmented ensemble docking, they finally identified 12 compounds with binding affinities less than 30 µM. In the work of Kohlhoff et al., cloud computing was used to simulate two milliseconds of GPCRs’ dynamics, which were then aggregated by MSMs to reveal multiple activation pathways [90]. Markov states identified from the high-flux pathways were docked against a database of β_2_AR agonists, antagonists, and decoys. The results show that using intermediate structures of β_2_AR identified by MSM analysis expands the chemical space to substances that could be missed by screening only a few structures of the receptor extracted from unbiased MD simulations.

To sum up, molecular docking has become one of the most frequently used methods in GPCR SBDD for its high-throughput nature. Homology modeling, as well as sequence-based modeling methods, is useful for acquiring experimentally inaccessible 3D structures of the receptor, while IFD and ensemble docking methods could further improve the efficiency of docking.

### 3.2. The Most Promising Methods: Free-Energy-Calculation Methods 

Although docking provides a useful tool to rapidly screen large compound libraries, it trades accuracy for speed. Its power to pick the real hits varies widely according to the receptor [91,92]. The approximate free energy methods, like LIE and MM-PBSA/GBSA, are physically more rigorous because they take the motion of the solvent and the receptor into account. They could be used in reordering the small amounts of compounds resulting from a large-scale docking campaign. There are also more accurate methods for predicting ligand–receptor binding affinity, such as thermodynamic integration (TI) [93], free energy perturbation (FEP) [94], metadynamics (MetaD) [16], etc. These approaches use MD simulations to extensively sample the conformational space and thus are more resource-consuming. In the past, it has been challenging to employ such accurate free energycalculation methods for relatively high-throughput screenings. Owing to the advances in both computing hardware and sampling algorithms, free energy calculations can now generate experimentally comparable results in a reasonable time frame, allowing its contribution to the lead optimization stage of drug discovery projects.

FEP, one of the most widely used methods, has been utilized in the validation of GPCRs’ homology models, the rationalization of the ligand–receptor binding affinity, and the demonstration of other thermodynamic properties of binding [95,96,97,98,99,100,101]. Recently, Schrödinger, a commercial software, implemented FEP+ as a highly accurate affinity prediction tool [102]. Lenselink et al. conducted a systematic characterization of the performance of FEP+ in predicting the binding free energies of congeneric ligands to four different GPCRs [103]. The calculated results showed a high correlation to the experimental data in terms of the ranking order of compounds. Moreover, they predicted the binding affinities of a series of designed compounds to A_2A_AR and subsequently synthesized four of them. The predicted values are within 1 kcal/mol of the experimental results and one of the designed compound’s affinity is increased tenfold compared to the original one. It is thus indicated that FEP+ may serve as a guide to real-life lead optimization projects. Significant progress has also been made on the application of absolute binding free energy methods in drug design. To accurately predict the free energy profile of ligand binding to GPCRs in an efficient way, Saleh et al. combined well-tempered MetaD with a funnel-like boundary to model the binding of 12 diverse ligands to five GPCRs [17]. The overall root-mean-square error of the predicted binding free energies compared to the experimental results is less than 1 kcal/mol. This work provides a generally applicable MetaD scheme for predicting the ligand–GPCR binding affinity.

In general, free-energy calculation methods, such as FEP and metadynamics, are now the fastest growing approaches and have proven effective in several GPCR-targeting drug design studies. With the evolution of the speed and algorithms, these accurate free-energy calculation methods would exert more significant influences on future drug discovery programs.

### 3.3. Case Study 2: Computer-Aided SBDD as a Useful Tool for Probing the Pharmacological Functions of Dark GPCRs 

**Figure 2:**
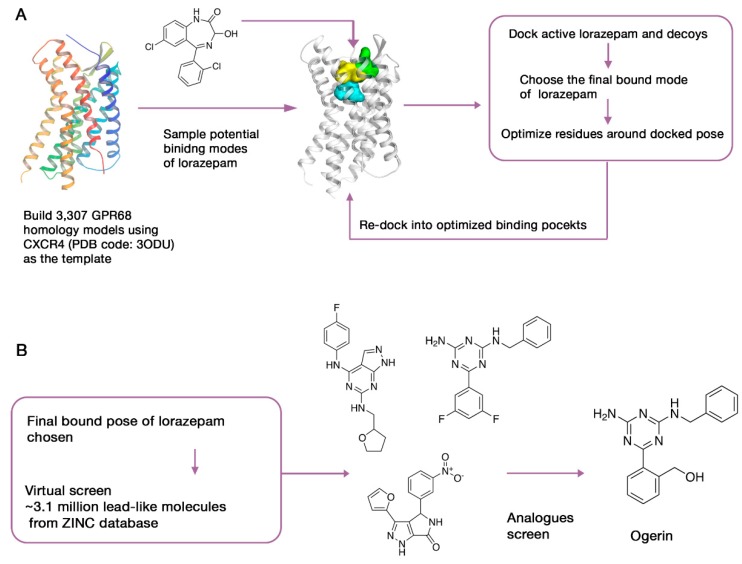
A virtual screening workflow for the discovery of probes targeting GPR68. (**A**) The acquisition of predicted lorazepam–GPR68 binding conformation through several cycles of optimization. (**B**) Virtual screen and analogues screen of ZINC database to identify hits with high affinity and selectivity.

Due to the significant roles of GPCRs in physiology and disease, understanding the biological functions of orphan GPCRs could lead to potential therapeutic benefits. Recently, SBDD has been combined with physical screening to demystify two pharmacologically dark GPCRs, GPR68 and GPR65 [104]. Just like kinases, proteases, and epigenetic proteins, the identification of ligands against orphan GPCRs provides new insights into their biological functions. Starting with yeast-based screens against GPR68, Huang et al. identified lorazepam as a putative positive allosteric modulator (PAM). The following tests of its analogues achieved poor improvements in activity. To further increase both the activity and the selectivity of ligands to GPR68, they conducted a large-scale virtual screening (Figure 2).

Due to the lack of a 3D structure of GPR68, 407 homology models were generated with the crystal structure of CXCR4 as the template. However, the sequence identity between the template and the target are too low (<29%) to acquire a reliable model. To increase the explored conformational space, they used elastic network modeling to sample the backbone and loop conformations of the crude models. The resulting 3307 models were then docked against the active and inactive compounds, including 440 from the National Clinical Collection (NCC) library and 176 property-matched decoy molecules generated by DUD-E [105]. Five putative allosteric sites of each model were tested, which corresponds to the binding pockets of the peptide and antagonist of CXCR4, the allosteric site of muscarinic receptor, and the orthosteric site of aminergic GPCRs. Next, iterative cycles of modeling and optimization were launched, aiming to reproduce the activity of lorazepam and its analogs as PAMs as well as the role of critical residues in the ligand-bound conformations. The cycle continued until all the top-ranked models converged to the same docking pose of lorazepam. 

As mentioned above, models generated using ligand-supported HM methods need to be improved and validated with substantial experimental information. In this work, mutagenesis combined with calcium release experiments was conducted on the critical residues inside the binding area to validate the predicted bound conformation of lorazepam. After that, approximately 3.1 million lead-like compounds from the ZINC database [106] were docked to the putative binding region in GPR68. Seventeen compounds were picked from the top 0.1% of the docked molecules for real biological tests. These chemically diverse compounds were selected for their high docking ranks and their ability to recap core interactions seen in the lorazepam–receptor complex. Four hits were found, and 25 of their analogues were then purchased and tested. Finally, 13 compounds with greater activity than lorazepam were identified, and their allosteric activities were confirmed. Ogerin, one of the identified hits with a strong allosteric effect and high specificity, was used as a probe to further investigate the downstream signaling and functional role of GPR68. By evaluating the effect of ogerin on GPR68-knockout and wild-type mice in a learning and memory test, the role of GPR68 in hippocampal-associated memory was revealed. 

To increase the usability of this approach, the same protocol was applied to another orphan receptor, GPR65. The success of these applications proves that computer-aided SBDD combined with experimental assays could serve as a useful tool for probing the new biological functions of understudied receptors, which constitute nearly 38% of non-olfactory GPCRs.

## 4. Summary and Perspectives

Computational modeling has grown into an integral part of the ligand–receptor recognition studies. Here, we have reviewed recent trends and applications of molecular modeling methods in investigating ligand–GPCR associations and drug discovery of GPCRs. Although the use of unbiased MD simulations in exploring the spontaneous binding of ligands to receptors is now possible, enhanced sampling techniques are more efficient to understand the comprehensive thermodynamic and kinetic aspects underlying the recognition. The research into GPCR-ligand binding pathways was once focused on the association of compounds directly from the water phase to the extracellular binding site. Not until recently has the importance of the membrane in the overall process been recognized, and membrane-involved binding mechanisms will still be an essential issue in the future. 

GPCRs participate in human pathophysiology and are now the most intensively investigated drug targets in pharmaceutical studies. As the target of more than 30% of marketed drugs, the identification of GPCRs’ ligands is of great pharmaceutical interest. Additionally, given the important role of GPCR in clinical therapeutics, the discovery of compounds binding to functionally unknown GPCRs could probably provide new areas of medical intervention. Molecular docking is currently the most commonly used computational method in GPCR-related SBDD programs due to its ability to predict receptor–ligand binding with relatively high throughput. HM or sequence-based modeling methods have proven effective at acquiring 3D models of GPCRs for use as the basis of docking. Another kind of computational SBDD method, the freeenergycalculation method, is growing fast and has shown significant potential for accurately evaluating limited number of compounds’ binding affinity to a receptor. With the evolution of methodologies along with computational hardware, it is expected that these approaches together can form an integrated computational platform with a robust capacity to identify desirable ligands against GPCRs. 

## Figures and Tables

**Table 1:** Examples of docking campaigns for the discovery of G protein-coupled receptors ligands.

Year/Reference	Target	Method ^1^	PDB Code	Known Active Ligand (s)	Receptor Models (Initial/Final)	Screened Compounds	Hit Rate (Hit/Tested)
2003/[52]	D3 dopamine receptor	HM	1f88	--	4	6727	0.55 (11/20)
2004/[53]	Neurokinin-1 (NK1) receptor	HM	1hzx	1	100/1	827,000	0.14 (1/7)
2004/[54]	5-HT_1A_ serotonin receptor	PREDICT	--	1	1	40,000	0.21 (16/78)
	NK1 receptor			1	1	150,000	0.15(5/53)
	5-HT_4_ serotonin receptor			1	1	150,000	0.21 (19/93)
2005/[55]	Alpha1A adrenergic receptor	HM	1f88	1	100/1	22,950	0.46 (37/80)
2007/[56]	CCR5 chemokine receptor	HM	1f88	5	--/1	1,620,316	0.17 (10/59)
2008/[57]	MCH-R1	HM	1l9h	4	20/1	187,084	0.05 (6/129)
2008/[58]	FFAR1	HM	1gzm	1	100/	2,600,000	0.29 (15/52)
2008/[59]	TRH-R1	HM	1f88	--	1	1,000,000	0.05 (5/100)
2009/[60]	β2 adrenergic receptor	X-ray	2rh1	--	--	1,000,000	0.24 (6/25)
2010/[61]	Adenosine A2A receptor	X-ray	3eml	--	--	4,000,000	0.41 (23/56)
2011/[62]	D3 dopamine receptor	HM	2vt4+2rh1	1300	20,000/1	3,300,000	0.23 (6/25)
		X-ray	3pbl	--	--	3,300,000	0.2 (5/25)
2015/[63]	Adenosine A2A receptor	X-ray	3qak/2ydo/2ydv	--	--	6,700,000	0.45 (9/20)
2016/[64]	μ-opioid receptor	X-ray	4dkl/5cm1	--	--	>3 million	0.30 (7/23)
2017/[65]	D4 dopamine receptor	X-ray	3pbl	--	--	>600,000	0.2 (2/10)
2017/[66]	MRGPRX2 opioid receptor	HM	4djh	1	1080	~3.7 million	0.05 (1/20)
2018/[67]	M2 mAChR	X-ray	3uon	--	--	4.6 million	0.23(3/13)

^1^ The method used in acquiring the receptor 3D structures for docking. HM: homology modeling. PREDICT program were used to generate 3D models of receptors in several docking studies. MCH-R1: Melanin-concentrating hormone receptor 1. FFAR1: Free fatty acid receptor 1. TRH-R1: Thyrotropin-releasing hormone receptor type 1. MRGPRX2: Mas-related G-protein coupled receptor member X2.
